# Weighted miRNA co-expression networks analysis identifies circulating miRNA predicting overall survival in hepatocellular carcinoma patients

**DOI:** 10.1038/s41598-020-75945-2

**Published:** 2020-11-03

**Authors:** Devis Pascut, Muhammad Yogi Pratama, Francesca Gilardi, Mauro Giuffrè, Lory Saveria Crocè, Claudio Tiribelli

**Affiliations:** 1 Liver Research Center, AREA Science Park, Fondazione Italiana Fegato-ONLUS, ss14, km 163.5, bldg. Q, Basovizza, 34149 Trieste, Italy; 2 Universitas Hasanuddin, Faculty of Medicine, Makassar, Indonesia; 3 Department of Medical Sciences, University of Trieste, Trieste, Italy; 4 Clinica Patologie Fegato, Azienda Sanitaria Universitaria Giuliano Isontina (ASUGI), Via Costantino Costantinides 2, Trieste, Italy

**Keywords:** Cancer, Computational biology and bioinformatics

## Abstract

The weighted gene co-expression network analysis (WGCNA) has been used to explore gene expression datasets by constructing biological networks based on the likelihood expression profile among genes. In recent years, WGCNA found application in biomarker discovery studies, including miRNA. Serum samples from 20 patients with hepatocellular carcinoma (HCC) were profiled through miRNA 3.0 gene array and miRNAs biomarker candidates were identified through WGCNA. Results were validated by qRT-PCR in 102 HCC serum samples collected at diagnosis. WGCNA identified 16 miRNA modules, nine of them were significantly associated with the clinical characteristics of the patient. The Red module had a significant negative correlation with patients Survival (− 0.59, p = 0.007) and albumin (− 0.52, p = 0.02), and a positive correlation with PCR (0.61, p = 0.004) and alpha-fetoprotein (0.51, p = 0.02). In the red module, 16 circulating miRNAs were significantly associated with patient survival. MiR-3185 and miR-4507 were identified as predictors of patient survival after the validation phase. At diagnosis, high expression of circulating miR-3185 and miR-4507 identifies patients with longer survival (HR 2.02, 95% CI 1.10–3.73, p = 0.0086, and HR of 1.75, 95% CI 1.02–3.02, p = 0.037, respectively). Thought a WGCNA we identified miR-3185 and miR-4507 as promising candidate biomarkers predicting a longer survival in HCC patients.

## Introduction

Hepatocellular carcinoma (HCC) is the fourth most common cause of cancer-related death worldwide^1^. It remains a “difficult to treat cancer”, especially in consideration of the heterogeneity of the disease. On this regards, the introduction of precision medicine opens new perspectives for the use of molecular classification models to stratify patients according to molecular determinants that may influence treatment protocols and survival outcomes. Several studies have documented the use of non-coding RNAS (ncRNAs) as potential prognostic biomarkers in cancer^2–4^, and microRNAs (miRNAs) represent the most widely investigatedclass of ncRNAs, in this regard. MiRNAs are short RNA molecule (18–25 nucleotide bases) participating in post-transcriptional regulatory mechanisms in cells^5^. MiRNAs can be released in extracellular compartments in which they are extraordinary stable being resistant to RNAses degradation, pH and temperature variations^6^. The discovery of circulating miRNAs opened new perspectives in the field of biomarker discovery, especially in consideration of their regulatory role in diseases^7^.

The increasing number of transcriptome and miRNome studies made available a multitude of complex-omic data, not always of easy interpretation. Thus new analytical tools, facilitating the interpretation of data, may be of particular interest to the scientific community.

Since its introduction, the weighted gene co-expression network analysis (WGCNA) has been used to explore gene expression datasets by constructing biological networks based on the likelihood expression profile among genes^8^. WGCNA groups genes that share common expression patterns and have a strong interconnection. These groups represent a sub-network region called “module”. The most representative genes of the module are summarized with the term “eigengene”, which is quantified as the overall gene expression level^8^. The rationale behind this procedure is the assumption that co-expressed genes might share similar functions, thus members from the same module are likely to participate in the same pathway. In addition WGCNA can link each module of co-expressed genes to a specific clinical variable of patients, thus providing a possible molecular mechanisms characterizing the disease. More recently, WGCNA has been used to identify circulating miRNA biomarkers in several morbid conditions, including cancer^9,10^, giving new hints for the application of this technique to miRNome studies.

In this study, we profiled circulating miRNAs from HCC patients, and the expression data were analyzed through a WGCNA to identify hub miRNAs as potential prognostic biomarker candidates. Relevant miRNAs were then validated through qRT-PCR in a larger cohort of patients to verify the potential predictive role of circulating miRNA candidates.

## Materials and methods

### Patients enrolment

One hundred and twenty-two consecutive patients referred to the Liver Clinic, Trieste University Hospital, Italy, between 2012 and 2017, with HCC diagnosed according to the EASL criteria, were enrolled in the study. Samples were collected at the time of HCC diagnosis, before any medical or surgical treatment.

All the patients provided written informed consent, and their clinical characteristics and collected blood samples were stored anonymously. The study was conducted prospectively and according to the criteria set by the declaration of Helsinki. In addition, it was formally approved by the regional Ethical Committee (Comitato Etico Regionale Unico FVG, Protocol Number 14/2012).

In the discovery phase, 20 patients were profiled through miRNA gene array, as previously described^11^. The clinical and demographic features of the groups are shown in Table S1. In the validation phase, 102 HCC patients were enrolled. Participants were predominantly male (80%) with a mean age of 70 (48–87 95% CI). The etiology of chronic liver disease was alcohol abuse and/or metabolic in the majority of HCC patients, while the viral component is present in 45% of the patient either alone or in combination with alcohol abuse and/or metabolic syndrome. The great majority of the patients were CTP score A (71.5%). HCC was staged according to the Barcelona Clinic Liver Cancer (BCLC) staging system into four groups, BCLC 0, A, B, C/D respectively in the 8%, 59%, 25%, and 6% of the patients. As for AFP level, 53% had AFP level lower than 20 ng/mL, 13% between 20 ng/mL and 400 ng/mL, and 10% with AFP level above 400 ng/mL. All clinical characteristics of the patients are reported in Table S1.

### Sample collection and miRNA Profiling

Fasting paired whole blood and serum samples were collected from the same participants during clinical visits. Serum was collected in Vacuette serum separating tubes (Greiner Bio-One GmbH, Austria). Samples were centrifuged at 3500 rpm for 10 min, aliquoted and stored at − 80 °C until further use.

Small RNAs were isolated from 300 μL of serum using the miRCURY RNA Isolation Kits (Exiqon, Vedbaek Denmark) and quantified with the Qubit microRNA Assay Kit (Thermo Fischer Scientific, USA). The quality of the total small non-coding RNAs extracted was assessed with the Agilent Small RNA kit (Agilent Technologies, USA), by using the 2100 Bioanalyzer Instrument (Agilent Technologies, USA). Small non-coding RNAs were hybridized on the Affimetrix array platform and analyzed as previously described^11^.

### Co-expression gene network analysis based on circulating miRNA data

The Weighted gene correlation network analysis (WGCNA) was used to build the circulating miRNA co-expression network according to the procedure of the WGCNA package in R language^8^. Firstly, the gene expression similarity matrix was constructed by calculating the absolute value of the Pearson’s correlation coefficient between gene pairs. Subsequently, the gene expression similarity matrix was converted into and an adjacency matrix by using a power adjacency function, which encodes the strength of the connection between node pairs. According to the scale-free topological algorithm, the adjacency matrix met the scale-free topology criterion when R^2^ value was approximated to 0.80.

The adjacency matrix was converted into a topological matrix, which uses the topological overlap measure (TOM) to describe the degree of association between miRNAs. TOM indicates the degree of dissimilarity between miRNAs pairs. The hierarchical clustering was constructed considering the 1-TOM as a distance, modules of co-expressed miRNAs were then identified by using the dynamic tree cut procedure. The minimum size cut-off of 5. Highly similar modules were merged with the function Merge Dynamic. The most representative group of miRNA for each module is named eigengenes (ME, module eigengene) or EigenmiRNA module (MEM), representing the overall level of miRNA expression in the module. The EigenmiRNA of a module is defined as the eigenvector associated with the first principal component of the expression matrix representing the expression profile of all miRNAs within a given module. For details in the procedure please refer to the works of Langfelder et al. and Zhang et al.^8,12^.

### Identification of hub genes

For the selection of miRNA biomarker candidates, modules associated with the clinical trait of interest, in our case ”survival”, were further considered for the intranodal analysis. The significance cut-off for the module selection was p = 0.05. Within the module, the gene significance (GS) and module membership (MM) were considered. GS is defined as the absolute value describing the relationship between the miRNA and the clinical trait, while the MM describes the correlation between the MEM and the miRNA expression profile. Thus miRNA candidates were searched among the top-scoring miRNAs according to MM and GS parameters. For details in the procedure please refer to the works of Langfelder et al. and Zhang et al.^8,12^.

### Validation of circulating miRNAs by qRT-PCR

Thirty nanograms of sncRNAs were reverse transcribed by using the qScript microRNA cDNA Synthesis Kit (Quantbio, Beverly, MA) according to manufacturer instruction. The RT-qPCR was performed with the PerfeCTa SYBR Green SuperMix (Quantbio, Beverly, MA) in a CFX-96 thermal cycler (Bio-Rad Laboratories, Hercules CA) according to manufacturer instructions. All reactions were run in duplicate in a 25 μL reaction. The relative quantification was obtained using the Pfaffl modification of the 2^−ΔΔCq^ equation, taking into account the efficiencies of individual genes, and results were normalized to miR-1280^13^.

### Data analysis and statistical methods

The Mann–Whitney U test was used to compare the difference between the two independent groups. Survival performance was estimated with the Kaplan–Meier methods. Analyses were performed by using NCSS 11 Software (2016) (NCSS, LLC. Kaysville, Utah, USA, ncss.com/software/ncss) and Stata 16.0 (Stata Corporation, College Station, TX, USA).

### Ethics approval and consent to participate

All the patients provided written informed consent and patient anonymity has been preserved. The investigation was conducted according to the principles expressed in the Declaration of Helsinki. The study was approved by the regional ethical committee (Comitato Etico Regionale Unico FVG, No. 14/2012 ASUITS).

## Results

### Construction of weighted co-expression network and identification of modules

Serum samples were profiled through miRNA 3.0 gene array and already described in-depth elsewhere^11^. According to the Absent/Present calling of the Affymetrix algorithm, we identified 274 miRNAs species expressed in serum. MiRNAs were further analyzed through WGCNA.

Before network construction and module detection samples were clustered and visualized in a heatmap to define how clinical traits relate to the sample dendrogram (Fig. S1).

**Figure 1:**
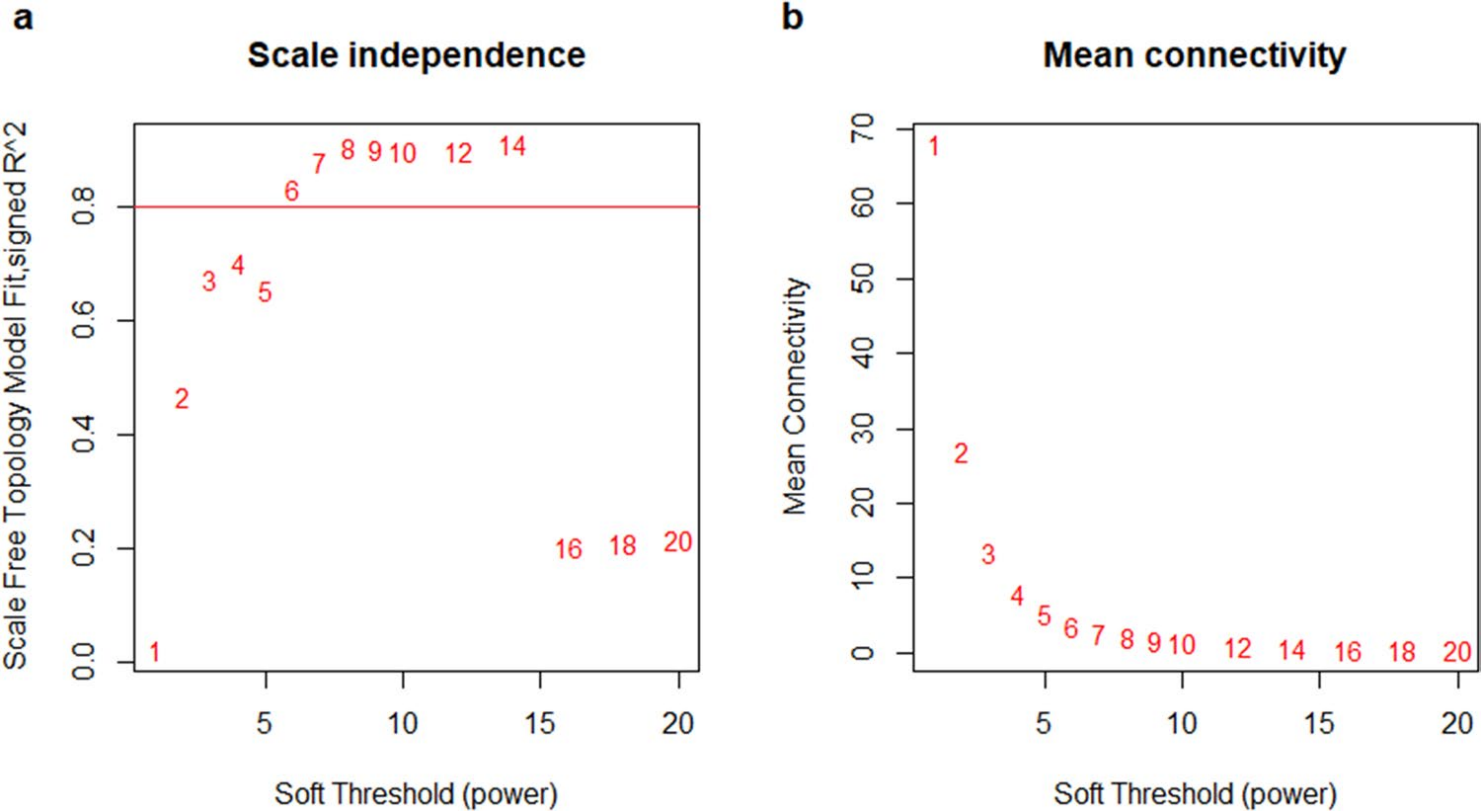
Determination of soft-thresholding power in the WGCNA. (**a**) The plot shows the scale-free topology fit index (y-axis) for different soft-thresholding powers (β) (x-axis). (**b**) Analysis of the mean connectivity (degree, y-axis) for various soft-thresholding powers (x-axis).

For network construction, the absolute value of the correlation of paired miRNAs was used to define the gene co-expression network. Subsequently, the gene expression similarity matrix was converted into and an adjacency matrix, based on the criterion of approximate scale-free topology, to define the strength between connected miRNAs. The soft thresholding power value was selected as β = 6 (scale-free R2 = 0.80) to ensure a scale-free network (Fig. 1a,b).

**Figure 2:**
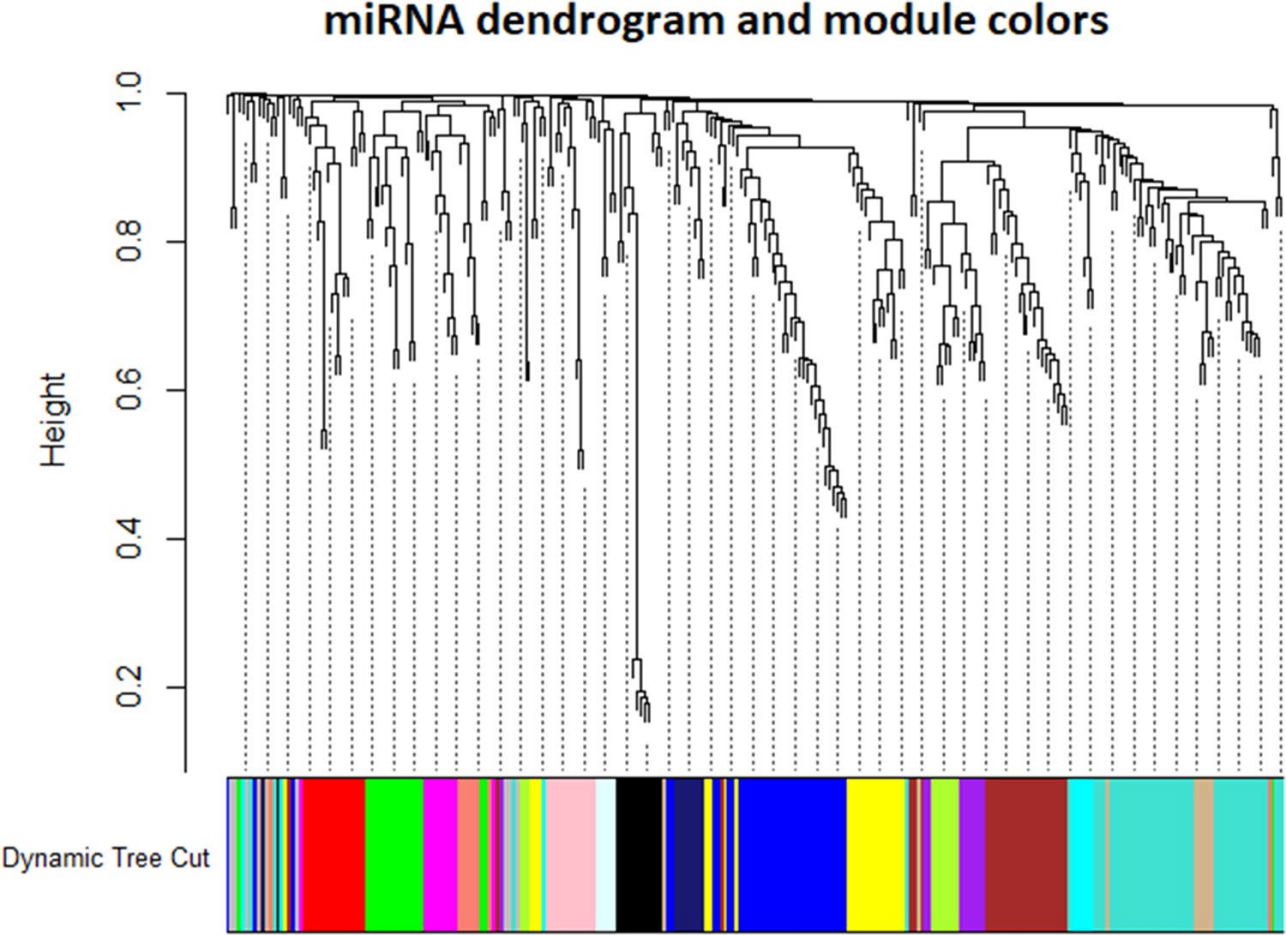
MiRNA dendrogram showing the co-expression modules. MiRNAs were clustered based on a dissimilarity measure (1-TOM). Correlated miRNA were grouped into colored modules identified through the Dynamic Tree Cut function. Each leaf, which is a short vertical line, corresponds to a specific miRNA. Branches of the dendrogram group together densely interconnected, highly co-expressed miRNAs.

The hierarchical agglomerative clustering was constructed considering the 1-TOM as a distance to identify the groups of co-expressed miRNAs. Modules of co-expressed miRNAs were then determined by using the dynamic tree cut procedure with a minimum module size cut-off of 5. This cut-off was chosen considering the miRNA transcriptome’s small size and the fact that a single miRNA can target multiple RNA transcripts. With this procedure, 18 different modules of EigenmiRNA were identified (Fig. 2).

**Figure 3:**
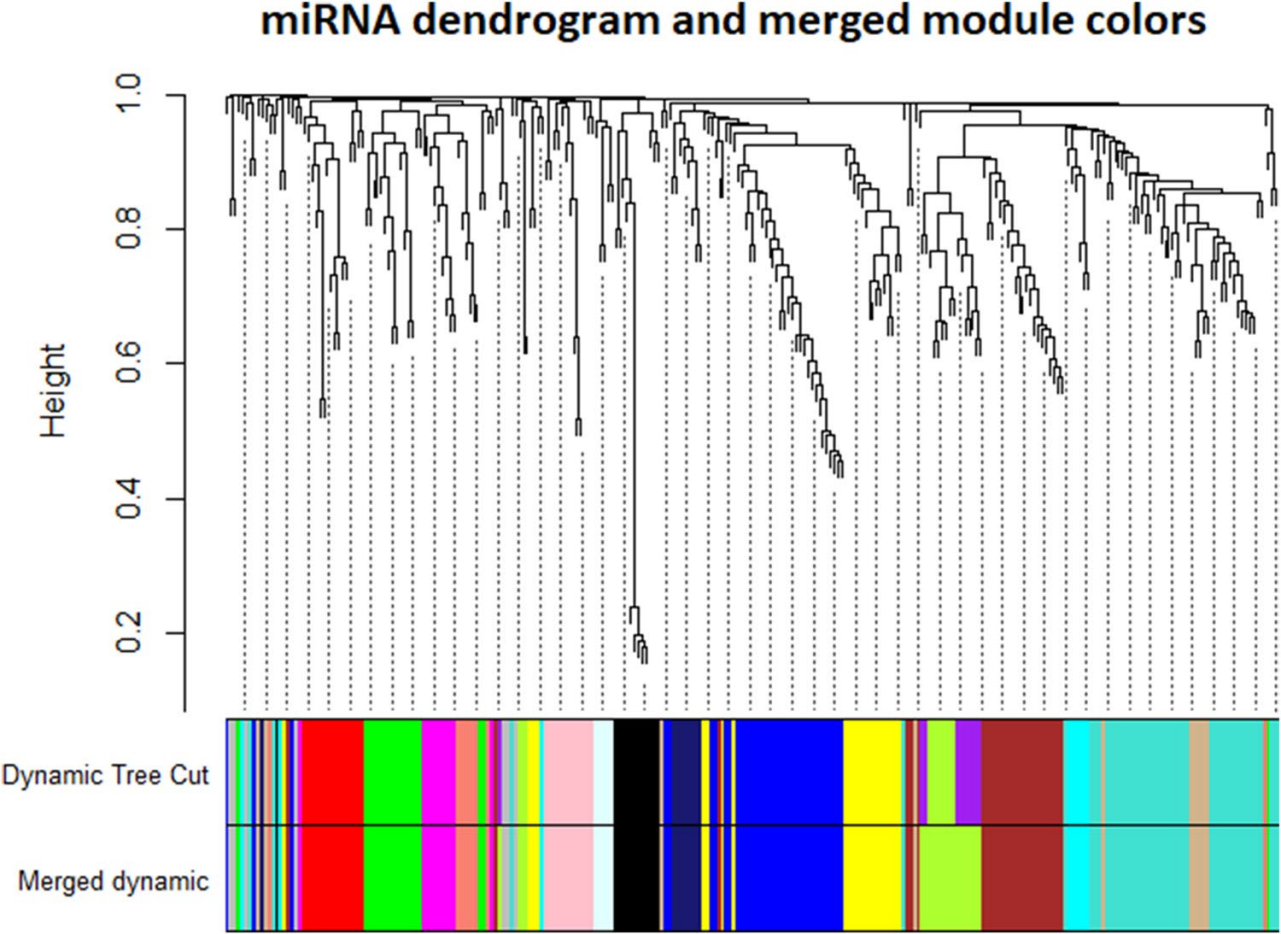
Merged miRNA modules. Highly correlated module colors were merged, identifying a total of 16 modules. Correlated miRNA were grouped into colored modules identified through the Dynamic Tree Cut function. Each leaf, which is a short vertical line, corresponds to a specific miRNA. Branches of the dendrogram group together densely interconnected, highly co-expressed miRNAs.

Modules were then clustered to identify modules with a similarity higher than 75% (Fig. S2). Similar modules were merged to identify a total of 16 modules, and miRNAs not classified in a correlated module were grouped in the grey module (Fig. 3). The size of miRNA modules ranges from a minimum of 5 miRNA of the Lightcyan module to the 46 miRNAs of the Turquoise module. Only seven miRNA were not classified in any of the colored modules and then included in the Grey one (Table S2).

### Correlation between modules and clinical traits

**Figure 4:**
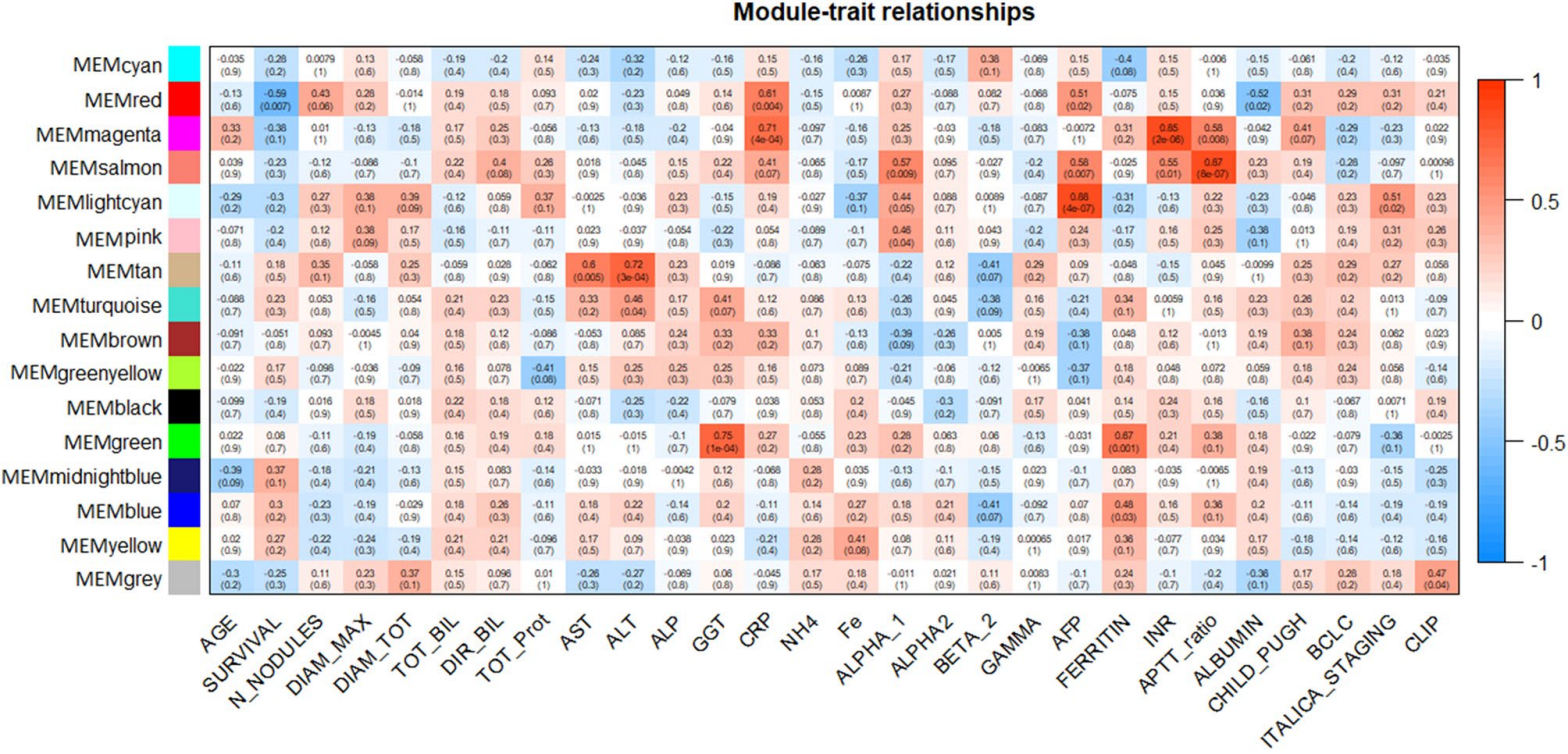
Relationships of consensus MEMs and clinical traits. Each row in the table corresponds to a module and each column to a clinical trait. Numbers in the table report the correlations scores between MEMs and traits, with the p-values of the correlations in parentheses. The table is color-coded by correlation according to the color legend on the right, Blue indicates a negative correlation while Red a positive one.

MEMs were then related to the clinical traits, including age, survival time, tumor nodule-related information, biochemical variables, tumor clinical scores (Child–Pugh, BCLC, ITALICA staging, Clip) (Fig. 4). Considering the correlation p-value cut off of 0.05, cyan, brown, green-yellow, black, midnight blue, yellow MEMs were not significantly associated with any clinical trait. Significant correlations identified for the remaining MEMs weresummarized in Table S3. While the majority of modules correlated with markers of liver function, such as transaminases, ferritin, and others, the red module positively correlates with the inflammation marker C-reactive protein (CRP) (r = 0.61; p = 0.004) and Alpha-fetoprotein (AFP) (r = 0.51; p = 0.02), and negatively with survival (r = − 0.59; p = 0.007) and albumin levels in serum (r = − 0.52; p = 0.02), thus suggesting the higher clinical relevance for miRNAs included into the red module. Interestingly, the red module also positively correlates with the number of tumor nodules (r = 0.43), although not reaching the statistical significance (p = 0.06) (Fig. 4).

### Modules associated with survival and predictive biomarker identification

**Figure 5:**
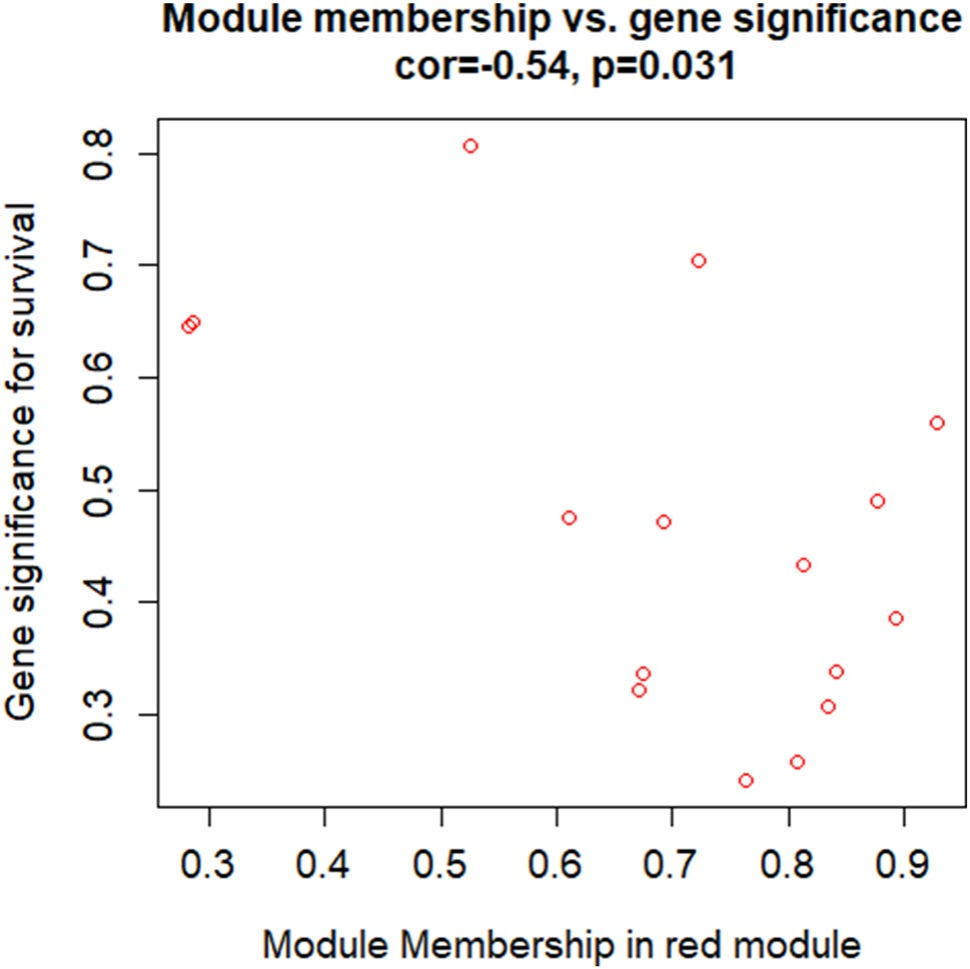
Intramodular analysis for MEM red. The figure shows the scatter plot of GS (y-axis) vs. MM (x-axis) for survival in MEM red. GS is the absolute value describing the relationship between the miRNA and the clinical trait, while the MM describes the correlation between the MEM and the miRNA expression profile.

Among all the significant correlations, we identified the association between MEM red and survival the most interesting from the clinical point of view. Consequently, we decided to analyze in-depth the MEM red to identify circulating predictive biomarkers associated with survival. Plotting the GS *vs.* MM for survival in the MEM red (Fig. 5), we found a significant negative correlation (r = − 54, p = 0.031) between miRNAs associated with survival (GS) and the MEM correlated to miRNA expression profiles (MM).

We ranked miRNAs included in the MEM red according to GS p-value. Within the MEM red. Eight miRNAs were significantly associated with survival, miR-3185, miR-4507, miR-125b-5p, and miR-30d-5p with a positive correlation, while miR-450b-5p, miR-603, miR-3646, miR-628-5p, with a negative one (Table S4). Other eight miRNAs resulted negatively correlated with survival, although they didn’t reach the statistical significance.

Although miR-125b-5p and miR-30d-5p have a significant correlation with survival, they have a low and not significant MM value for MEM red. MiR-450b-5p and miR-603 have the highest significant MM.

### Validation of biomarker candidates for survival in cancer patients

**Figure 6:**
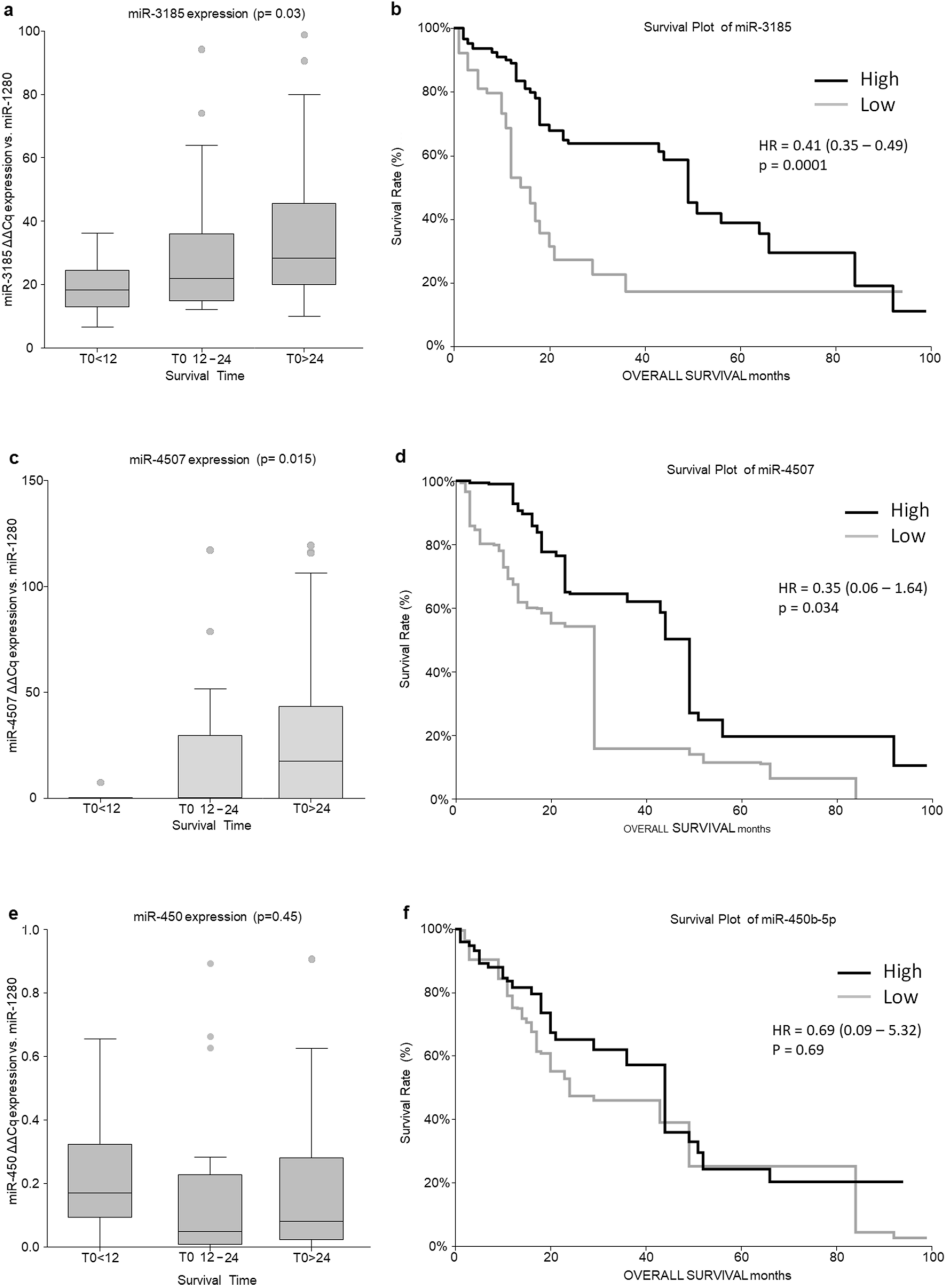
The differential expression of miR-3185 (**a**), miR-4507 (**c**) and miR-450b-5p (**e**) between patients having OS < 12, OS 12–24 and OS > 24 at T0. Kaplan–Meier survival analysis by log-rank test comparing high vs. low expression of miR-3185 (**b**), miR-4507 (**d**), and miR-450b-5p (**f**).

In the validation phase, we considered the top scoring miRNAs associated with survival. In particular, we considered the top two positively correlated (miR-3185, miR-4507) and the top two negatively correlated (mir-450b-5p, miR-603) miRNAs for further validation in 102 serum samples of HCC patients before treatments. Patients were divided into three groups, according to the survival time (months): OS < 12, OS 12–24, and OS > 24. We observed a significantly different expression of miR-3185 (p = 0.03) and miR-4507 (p = 0.015) (Fig. 6a,c). Serum miR-3185 levels gradually increase according to the longer OS, being 1.6 fold times higher in OS 12–24 months compared to OS < 12 months and 1.9 times higher in OS > 24 months compared to OS < 12 months (Fig. 6a and Table S5). The mean ΔΔCq expression of miR-4507 was extremely low in subjects with OS < 12 months, showing 29 times and 45 times increase in patients with OS 12–24 and OS > 24 months, respectively (Fig. 6c and Table S5). In the validation phase, miR-450b-5p was not statistically significant among the different populations (p = 0.45) (Fig. 6e,f), while miR-603 resulted undetectable at the considered working conditions.

To further investigate the potential of miRNA candidates as predictor of OS, in HCC patients before initiation of therapy, a Kaplan–Meier survival analysis was performed comparingpatients with high *vs.* low miR expression, using the mean of the low expression group as a cut-off. The lower expression of mir-4507 and miR-3185 was significantly associated with shorter OS at T0 with an HR of 0.35 (95% CI 0.06–1.64, p = 0.034) (Fig. 6b) and HR of 0.41 (95% CI 0.35–0.49, p = 0.0001), (Fig. 6d), respectively. The highest difference between the two groups of patients was maintained up to 4 years. Again miR-450b-5p resulted not significantly associated with OS (Fig. 6f).

## Discussion

Despite the recent advances in treatments and follow-up protocols, HCC still represents one of the deadliest cancer worldwide. The lack of reliable diagnostic and/or prognostic biomarkers severely limits patient survival. The availability of predictive models might improve clinical decisions resulting in better patient management. In recent years, WGCNA became an available and reliable method to identify hub genes involved in cancer pathways, including in HCC. Li et al. explored the Cancer Genome Atlas database (TGCA) to identify Hub genes involved in key tumorigenic pathways^14^. Similarly, Yin et al.^15^ analyzed peripheral blood mononuclear cells (PBMC)-derived RNA to identify diagnostic blood-based biomarkers for HCC. More recently, Li et al. found a six-gene signature that predicts OS in patients with HCC, however using tissue expression data. Although WGCNA was used to identify circulating miRNA biomarkers in other cancers^9,16^, no similar studies are available on HCC. In this study, we conducted a WGCNA on a miRNA array dataset to identify circulating miRNA associated with relevant clinical variables at the time of diagnosis. We identified the MEM Red inversely correlated to survival and albumin concentration, while positively correlated to CRP and AFP concentration. Interestingly this module correlated also with the nodules number, although with a p-value near to statistical significance (p = 0.06), suggesting a close relation with HCC. Since the identification of biomarkers able to predict the survival of HCC patients is still an unmet clinical need, we focus on MEM red to identify miRNA associated with this clinical variable. Among the selected miRNA candidates that were validated in 102 HCC serum samples, we identified miR-3185 and miR-4507 as significantly associated with survival. Patients having a high expression of miR-3185 and miR-4507 at diagnosis showed a longer OS, with miR-3185 showing the best performance in discriminating patients living longer. Despite these findings, it is hard to identify a clear link with HCC and the two miRNAs, since the lack of information in the current literature. MiR-3185 it is a generally low expressed miRNA (https://ccb-web.cs.uni-saarland.de/tissueatlas/patterns), whose presence was mainly identified in prostate adenocarcinoma (https://mirgator.kobic.re.kr/requestResult.html). However, other evidence linked this miRNA to the p53 regulatory network. TP53 missense mutation that impairs tumor suppression also causes the oncogenic gain of function with the downregulation of several miRNAs including miR-3185 in Gastric cancer^17^. The mir-target enrichment analysis associated this miRNA with several pathways related to cancer. However, none of the targets were validated in vitro, giving new opportunities to study in-depth the role of this miRNA in livers cancer (see “Supporting Material”, Figs. S3 and S4). The overall expression of miR-4507 is higher than miR-3185 with the liver being one of the highest mir-4507-expressing organs, together with colon and Kidney (https://ccb-web.cs.uni-saarland.de/tissueatlas). Interestingly other studies identified a progressive decrease of miR-4507 expression from chronic hepatitis B to Liver cirrhosis and HCC in the whole blood^18^, even if the precise function of this miRNA remains still unknown. Recent studies linked mir-4507 to the TGF-β pathway in cancer, however, similarly to miR-3185, no direct proof of this association was supporting the findings^19^.

Despite the limitations in the general knowledge about the role of miR-3185 and miR-4507 in cancer, we evidenced their potential as circulating biomarkers predicting longed survival in patients with HCC. WGCNA represents a useful tool also in miRNA biomarker discovery, giving new opportunities to shed light on complex regulatory networks that involve miRNAs in cancer.

To further promote the use of these two miRNA in a clinical setting, more extensive validating studies are needed, without forgetting to clarify the unexplored role of the two miRNA in cancer.

## Supplementary information

Supplementary Information 1.

Supplementary Information 2.

## Data Availability

The datasets used during the current study are available from the corresponding author on a reasonable request.
