# Supplementary material for: Weighted miRNA co-expression networks analysis identifies circulating miRNA predicting overall survival in hepatocellular carcinoma patients
Source: Sci Rep. 2020 Nov 3;10:18967. doi: 10.1038/s41598-020-75945-2 (PMC7609726; doi:10.1038/s41598-020-75945-2)
Supplement: Supplementary file 2 — Supplementary Information 2. [file 41598_2020_75945_MOESM2_ESM.docx]

Weighted miRNA co-expression networks analysis identifies circulating miRNA predicting overall survival in hepatocellular carcinoma patients

**Devis Pascut^1^, Muhammad Yogi Pratama^1,2^, Francesca Gilardi^1^, Mauro Giuffrè^1,3^, Lory Saveria Crocè^1,3^, Claudio Tiribelli^1^**

1. Fondazione Italiana Fegato - ONLUS, Liver Research Center, AREA Science Park, Basovizza, Trieste, Italy.
2. Universitas Hasanuddin, Faculty of Medicine, Makassar, Indonesia.
3. Department of Medical Sciences, University of Trieste, Trieste, Italy

Clinica Patologie Fegato, Azienda Sanitaria Universitaria Giuliano Isontina (ASUGI), Via Costantino Costantinides 2, Trieste, Italy

*** Corresponding author**

Devis Pascut

Fondazione Italiana Fegato - ONLUS

Area Science Park, ss14, km 163.5,

bldg. Q, 34149 Basovizza, Trieste

[devis.pascut@fegato.it](mailto:devis.pascut@fegato.it)

**Running title:** WGCNA identifies miRNA biomarkers predicting HCC patients’ survival

**Keywords:** microRNA, Biomarkers, WGCNA, HCC, Survival

**Financial Support**

The study was partially supported by the project surface-enhanced Raman microRNA for cancer (SERMI4CANCER) POR FESR 2014 2020 Friuli Venezia Giulia decree no.3028 05/05/2017 and no.4526 16/06/2017, Indonesia Endowment Fund for Education (LPDP) and an intramural grant from the Italian Liver

**Competing of interest statement:** The authors declare no conflict of interest.

**Word count:** 2997

**Number of Figures and Tables:** 6

**Supplementary materials and methods**

**Enrichment Analysis**

Mirna targets were searched in miRTarBase (<http://mirtarbase.mbc.nctu.edu.tw/php/index.php>)[1] release 7.0. Predicted Targets were searched in miRDB database(<http://mirdb.org>)[2]. Target enrichment analysis was performed pooling the results from miRTarBase and miRDB search by running the GeneCodis online tool ( <https://genecodis.genyo.es/>)[3] on KEGG Pathway database with default settings.

**Mir-Target enrichment analysis**

In miRDB 272 and 925 transcripts were predicted as possible miR-4507 and miR-3185 targets, respectively (File S1). While 66 and 166 transcripts were associated to miR-4507 and miR-3185, respectively, in miRTarBase. None of the two miRNA has strongly validated targets, the only data available in miRTarBase, consists of weekly associated targets. The target enrichment analysis assigned a role in cancer for both miRNAs (Fig. S3 and Fig. S4). MiR-3185 targets were enriched in 36 pathways, with the cancer-related pathways being the top scoring ones (Fig. S3), while mir-4507 targets were significantly enriched in 7 pathways, 5 of them related to cancer (Fig. S4)


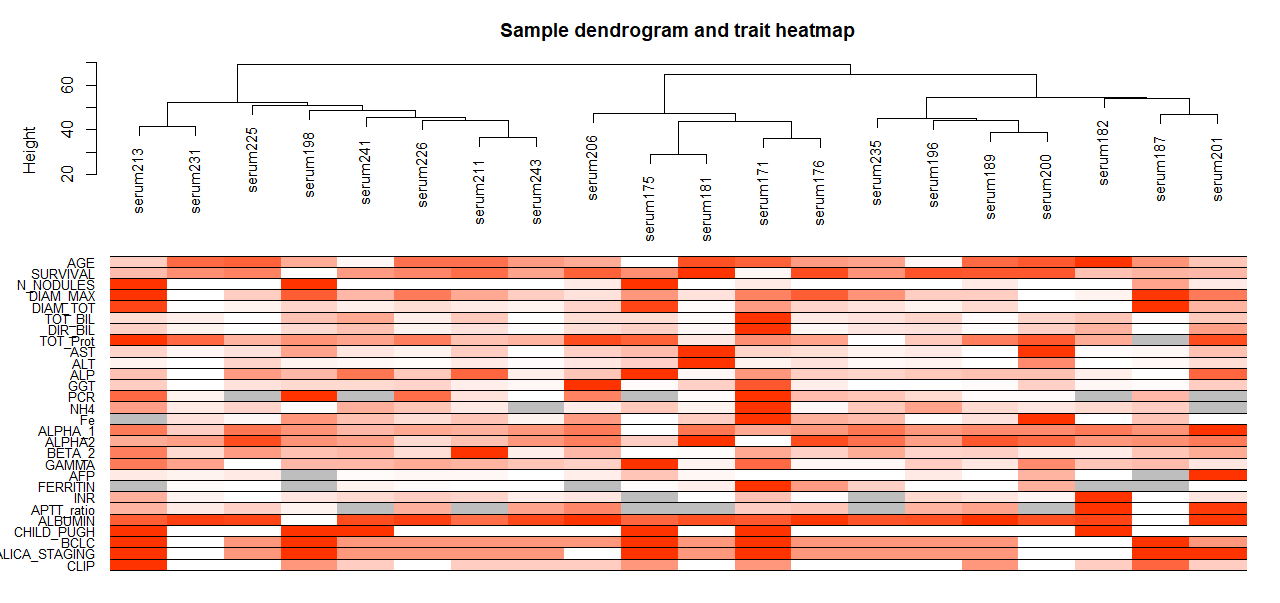


**Figure S1.** Clustering dendrogram of samples based on their Euclidean distance and heatmap of the clinical variables associated with each sample


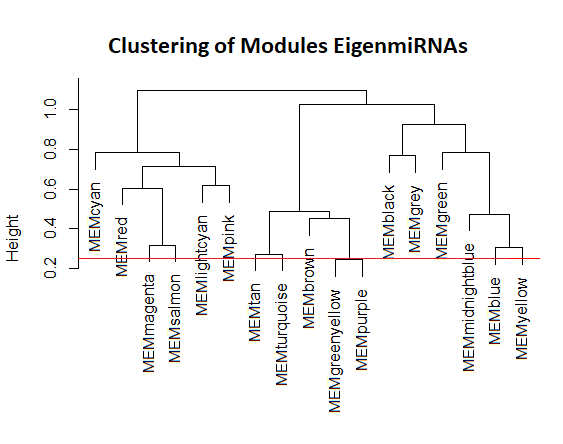


**Figure S2**. Clustering of the Modules EigenmiRNAs. The cluster merge height was set at 0.25


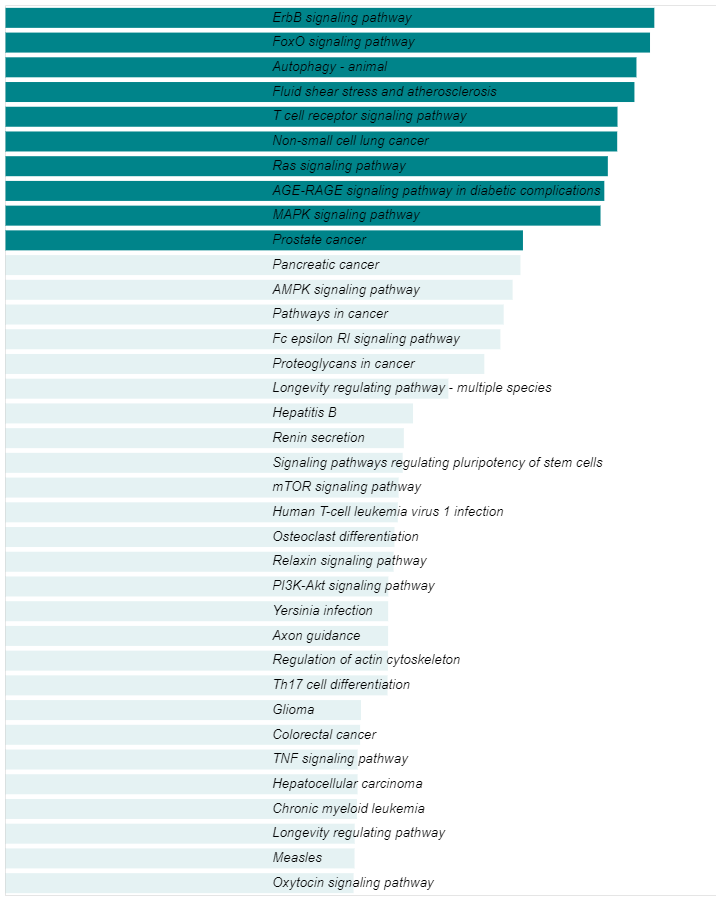


**Figure S3.** Bars-Chart of the 36 enriched pathways related to miR-3185 targets. The enrichment analysis was performed by using the Fisher’s exact test with a Hypergeometric distribution corrected for multiple testing by using the False Discovery Rate (FDR) correction


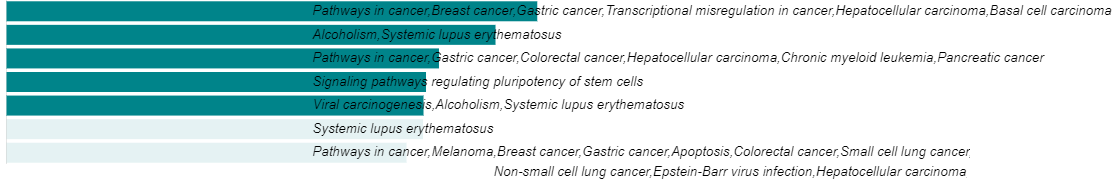


**Figure S4.** Bars-Chart of 7 enriched pathways related to miR-4507 targets. The enrichment analysis was performed by using the Fisher’s exact test with a Hypergeometric distribution corrected for multiple testing by using the False Discovery Rate (FDR) correction

**Table S1.** Demographic and clinical characteristics of the studied populations

|  | **Training Cohort (n=20)** | **Validation Cohort (n=102)** | |
| --- | --- | --- | --- |
| **Patients characteristics** |  | |  |
| Age (mean, 95%CI) | 68 (64-71) | | 70 (48-87) |
| Sex (M/F) | 17/3 | | 81/ 21 |
| **Aetiology** |  | |  |
| Alcohol metabolic | 16 | | 56 |
| Alcohol metabolic viral |  | | 10 |
| Viral | 4 | | 36 |
| **Disease scores** |  | |  |
| CTP A/B/C | 14/6/0 | | 73/22/3 |
| BCLC 0/A/B/C-D | 3/12/5/0 | | 8 / 61 / 25 / 6 |
| **Alpha fetoprotein** |  | |  |
| <20 ng/mL | 15 | | 54 |
| 20 - 400 ng/mL | 5 | | 14 |
| >400 ng/mL | 0 | | 10 |

**Table S2.** Number of miRNAs included in each module.

| **Module** | **N° of miRNAs** |
| --- | --- |
| Black | 13 |
| Blue | 37 |
| Brown | 27 |
| Cyan | 9 |
| Green | 19 |
| Greenyellow | 10 |
| Lightcyan | 5 |
| Magenta | 11 |
| Midnightblue | 9 |
| Pink | 13 |
| Purple | 10 |
| Red | 16 |
| Salmon | 9 |
| Tan | 10 |
| Turquoise | 46 |
| Yellow | 23 |
| Grey | 7 |

**Table S3.** Clinical traits significantly associated to MEMs

| **MEM color** | **Significant positive correlation** | **Significant Negative correlation** |
| --- | --- | --- |
| Red | CRP, AFP | Survival, Albumin |
| Magenta | CRP, INR, APTT ratio |  |
| Salmon | Alpha 1 , AFP, INR, APTT ratio |  |
| Lightcyan | Alpha 1, AFP, ITALICA staging |  |
| Pink | Alpha1 |  |
| Tan | ALT, AST | Beta 2 |
| Turquoise | ALT |  |
| Green | GGT, Ferritin |  |
| Blue | Ferritin |  |
| Grey | Clip score. |  |

**Table S4.** MiRNA included in MEM red ranked based on GS p value

| **miRNAs in MEM Red** | **GS.survival** | **p.GS.survival** | **MM.red** | **p.MM.red** |
| --- | --- | --- | --- | --- |
| miR-3185 | 0.81 | 1.69E-05 | -0.52 | 0.018 |
| miR-4507 | 0.70 | 0.001 | -0.72 | 0.001 |
| miR-125b-5p | 0.65 | 0.002 | -0.29 | 0.222 |
| miR-30d-5p | 0.65 | 0.002 | -0.28 | 0.229 |
| miR-450b-5p | -0.56 | 0.010 | 0.93 | 3.84E-09 |
| miR-603 | -0.49 | 0.028 | 0.88 | 3.77E-07 |
| miR-3646 | -0.48 | 0.034 | 0.61 | 0.004 |
| miR-628-5p | -0.47 | 0.035 | 0.69 | 0.001 |
| miR-570 | -0.43 | 0.057 | 0.81 | 1.28E-05 |
| miR-4423-3p | -0.39 | 0.093 | 0.89 | 1.19E-07 |
| mir-548aa | -0.34 | 0.144 | 0.84 | 3.33E-06 |
| miR-548aj-3p | -0.34 | 0.147 | 0.67 | 0.001 |
| miR-4529-3p | -0.32 | 0.166 | 0.67 | 0.001 |
| miR-19a-3p | -0.31 | 0.187 | 0.83 | 4.86E-06 |
| miR-3128 | -0.26 | 0.274 | 0.81 | 1.64E-05 |
| miR-3201 | -0.24 | 0.306 | 0.76 | 9.25E-05 |

**Table S5.** Circulating miRNA expression

|  | **Survival time (mean 95%CI)** | | | |
| --- | --- | --- | --- | --- |
| **miRNA** | **<12 months** | **12 – 24 months** | **>24 months** | **p value** |
| **miR-3185** | 18.61 (13.92-23.31) | 29.88 (21.27-38.49) | 35.68 (28.42-42.94) | p=0.03 |
| **miR-4507** | 0.71 (-0.67-2.09) | 20.99 (9.50-32.46) | 32.22 (20.47-43.96) | p=0.014 |
| **miR-450** | 0.21 (0.11-0.33) | 0.15 (0.056-0.24) | 0.17 (0.10-0.23) | p=0.6 |

**References**

1 Chou C-H, Shrestha S, Yang C-D *et al.* miRTarBase update 2018: a resource for experimentally validated microRNA-target interactions. *Nucleic Acids Res.* 46(D1), D296–D302 (2018).

2 Chen Y, Wang X. miRDB: an online database for prediction of functional microRNA targets. *Nucleic Acids Res.* 48(D1), D127–D131 (2020).

3 Tabas-Madrid D, Nogales-Cadenas R, Pascual-Montano A. GeneCodis3: a non-redundant and modular enrichment analysis tool for functional genomics. *Nucleic Acids Res.* 40(Web Server issue), W478-483 (2012).

**File S1.** List of the miR-4507 and miR-3185 predicted targets
